# Association of *LEP* G2548A and *LEPR* Q223R Polymorphisms with Cancer Susceptibility: Evidence from a Meta-Analysis

**DOI:** 10.1371/journal.pone.0075135

**Published:** 2013-10-17

**Authors:** Jing He, Bo Xi, Rikje Ruiter, Ting-Yan Shi, Mei-Ling Zhu, Meng-Yun Wang, Qiao-Xin Li, Xiao-Yan Zhou, Li-Xin Qiu, Qing-Yi Wei

**Affiliations:** 1 Cancer Institute, Fudan University Shanghai Cancer Center, Shanghai, China; 2 Department of Oncology, Shanghai Medical College, Fudan University, Shanghai, China; 3 Institute of Maternal and Child Health Care, School of Public Health, Shandong University, Jinan, China; 4 Department of Epidemiology, Erasmus MC, Rotterdam, The Netherlands; 5 Department of Pathology, Fudan University Shanghai Cancer Center, Shanghai, China; 6 Department of Medical Oncology, Fudan University Shanghai Cancer Center, Shanghai, China; 7 Department of Epidemiology, The University of Texas MD Anderson Cancer Center, Houston, Texas, United States of America; Central China Normal University, China

## Abstract

**Background:**

Numerous epidemiological studies have examined associations of genetic variations in *LEP* (G2548A, -2548 nucleotide upstream of the ATG start site) and *LEPR* (Q223R, nonsynonymous SNP in exon 6) with cancer susceptibility; however, the findings are inconsistent. Therefore, we performed a meta-analysis to comprehensively evaluate such associations.

**Methods:**

We searched published literature from MEDLINE, EMBASE, Web of Science and CBM for eligible publications. We also assessed genotype-based mRNA expression data from HapMap for rs7799039 (G2548A) and rs1137101 (Q223R) in normal cell lines derived from 270 subjects with different ethnicities.

**Results:**

The final analysis included 16 published studies of 6569 cases and 8405 controls for the *LEP* G2548A and 19 studies of 7504 cases and 9581 controls for the *LEPR* Q223R. Overall, *LEP* G2548A was statistically significantly associated with an increased risk of overall cancer (AA vs. GG: OR=1.27, 95% CI=1.05-1.54; recessive model: OR=1.19, 95% CI=1.00-1.41). Further stratifications by cancer type showed an increased risk for prostate cancer (recessive model: OR=1.26, 95% CI=1.05-1.51) but not for other cancers. For *LEPR* Q223R, no statistical evidence for an association with risk of cancer was found for all; however, further stratification by ethnicity showed an increased risk for Africans but not for other ethnicities. No significantly differences in *LEP* and *LEPR* mRNA expression were found among genotypes or by ethnicity.

**Conclusions:**

Despite some limitations, this meta-analysis found some statistical evidence for an association between the *LEP* 2548AA genotype and overall risk of cancer, particularly for prostate cancer, but given this variant did not have an effect on mRNA expression, this association warrants additional validation in large and well-designed studies.

## Introduction

Cancer is recognized as one of the leading causes of death in economically developed countries as well as in developing countries. With the estimated 12.7 millions of cancer cases and 7.6 millions of cancer deaths occurred in 2008, cancer has become a major public health challenge [1]. Because of the combination of earlier detection and improved treatment, the overall cancer mortality is decreasing over the last decade. However, the global burden of cancer continues to increase, largely due to the increased longevity and subsequent growth of the world populations that increasingly adopt cancer-causing behaviors [1]. While the mechanism of carcinogenesis is still not fully understood, it has been suggested that environmental factors, interplaying with low-penetrance susceptibility genes, may be important in the development of cancer [2,3].

**Figure 1:**
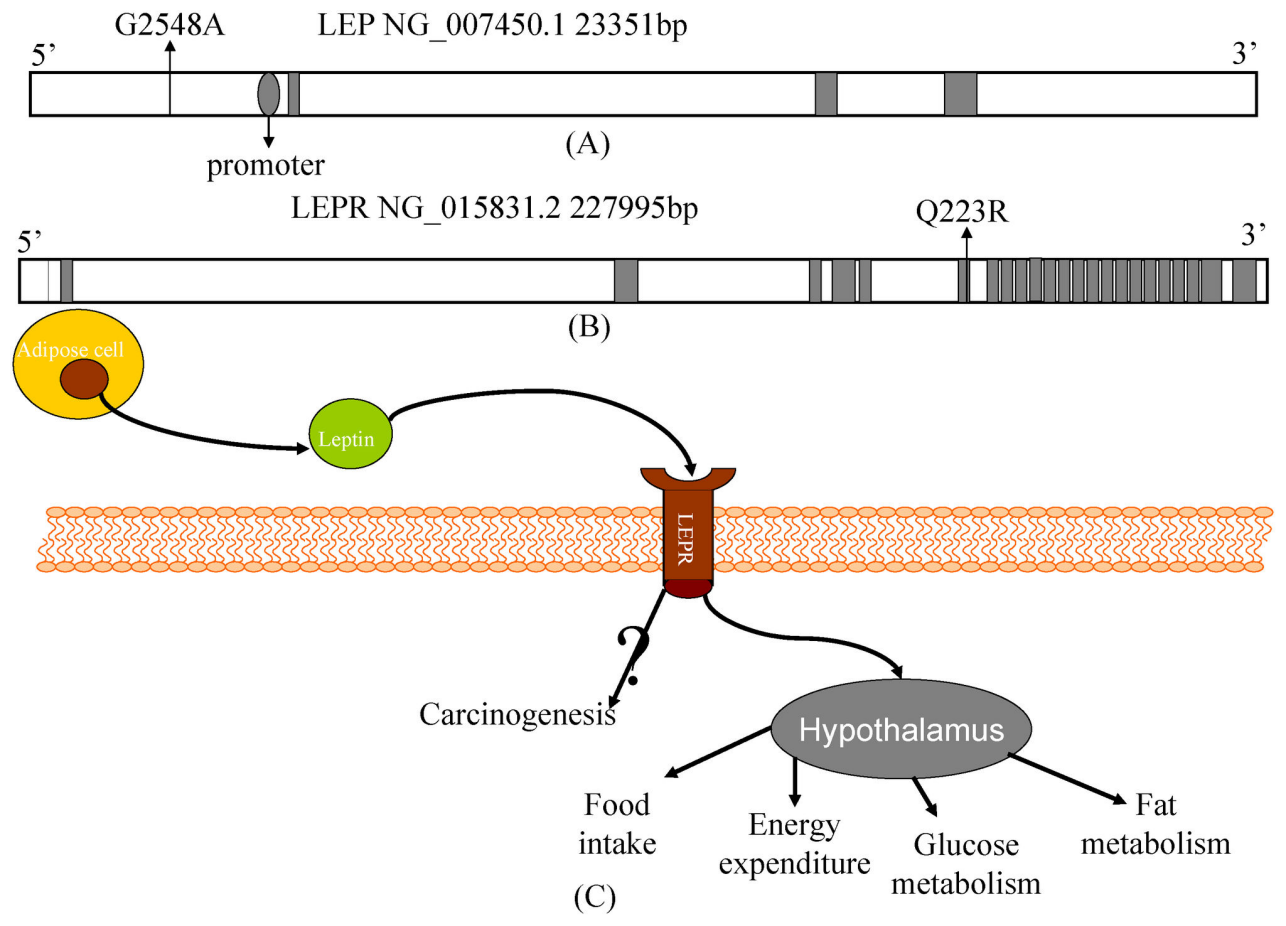
Gene structural characteristics of leptin and leptin receptor and their roles in regulating adipose tissue mass. The locations of *LEP* G2548A (A) and *LEPR* Q223R (B) with possible leptin functions and the pathway of regulating adipose tissue mass (C).

Leptin (LEP, also called OB for obese), an adipocyte-derived hormone, produced predominantly by white adipose tissue, regulates appetite and weight, body metabolism and reproductive functions together with the leptin receptor (LEPR) (Figure **1**) [4]. The *LEP* gene, located at chromosome 7q31.3, encodes a 16 kDa protein that has been consistently shown to be associated with endocrinologic metabolism [5]. It has been also suggested that leptin could contribute to serum insulin levels and the development of type 2 diabetes [6] and that leptin is involved in the pathophysiology of obesity [7,8] and carcinogenesis [9-14]. Leptin exerts its physiological action through its receptor (LEPR, also called CD295, and its gene is located at chromosome 1p31), which is a single transmembrane protein distributed in many types of tissues [15].

*LEP* and *LEPR* are highly polymorphic, and a number of single nucleotide polymorphisms (SNPs) have been identified in these two genes [6,8,16,17]. For example, there are at least 383 reported SNPs in the *LEP* gene region and 3117 reported SNPs in the *LEPR* gene region (http://www.ncbi.nlm.nih.gov/projects/SNP). However, only few of these reported SNPs are potentially functional and ever studied for their associations with cancer susceptibility. For *LEP*, there are two SNPs that reportedly change amino acid of the protein but only G2548A (rs7799039) was extensively investigated for its association with cancer risk; for *LEPR*, there are five common (minor allele frequency > 0.05) SNPs that may cause amino acid changes, but only Q223R (rs1137101) was studied for its association with cancer susceptibility. Because the results from these studies are inconsistent [9-14,16,18-40], we performed a meta-analysis of the published reports to further evaluate the association of these two SNPs with the risk of cancer.

## Materials and Methods

### Identification and eligibility of relevant studies

Published studies were included, if they met the following three inclusion criteria: (a) evaluating the association between *LEP* G2548A (or A19G) and/or *LEPR* Q223R SNPs and cancer risk, (b) using a case-control design, (c) providing sufficient data for calculation of an odds ratio (OR) with 95% confidence interval (CI).

We searched electronic literature MEDLINE, EMBASE, Web of Science and Chinese Biomedical (CBM) (http://www.imicams.ac.cn) databases for all relevant articles using the search terms: “leptin or LEP”, “leptin receptor gene or LEPR”, “variant, variation or polymorphism” and “cancer, carcinoma or tumor” (the last search was updated on March 10, 2013). All eligible studies were retrieved, and their bibliographies were manually checked for other relevant publications. Review articles and bibliographies of other relevant studies identified were hand-searched as well to find additional eligible studies. Only published studies with full-text articles in English or Chinese were included. If more than one article was published using the same patient population, only the latest or the largest study was used in this meta-analysis. Two authors independently assessed the articles for compliance with the inclusion criteria, and disagreement was resolved by discussions until the consensus was reached.

### Data extraction

The following information was collected from each study: first author's surname, publication date, ethnicity of the study population, cancer type, source used for controls, total number of cases and controls, and numbers of cases and controls with the AA, AG and GG genotypes for *LEP* G2548A and *LEPR* Q223R, respectively.

### Genotype and gene expression correlation analysis

The data on *LEP* and *LEPR* genotype and transcript (mRNA) expression levels were available online (http://app3.titan.uio.no/biotools/help.php?app=snpexp) [41]. The genotyping data were derived from The HapMap phase II release 23 data set consisting of 3.96 million SNP genotypes from 270 individuals from four populations (CEU: 90 Utah residents with ancestry from northern and western Europe; CHB: 45 unrelated Han Chinese in Beijing; JPT: 45 unrelated Japanese in Tokyo; YRI: 90 Yoruba in Ibadan, Nigeria) [42,43]. The transcript (mRNA) expression data by genotypes were from EBV-transformed B lymphoblastoid cell lines from the same 270 individuals [44,45].

### Statistical methods

The strength of associations of *LEP* G2548A and *LEPR* Q223R SNPs with cancer risk was assessed by calculating ORs with the corresponding 95% CIs. For *LEP* G2548A, the pooled ORs were performed for homozygous model (AA vs. GG), heterozygous model (AG vs. GG), recessive model (AA vs. AG+GG), and dominant model (AA+AG vs. GG). For *LEPR* Q223R, the pooled ORs were also performed for homozygous model (GG vs. AA), heterozygous model (AG vs. AA), recessive model (GG vs. AG+AA), and dominant model (GG+AG vs. AA). The homogeneity assumption was verified by using a Chi square-based Q-test. If the studies were found to be homogeneous (with *P*>0.10 for the Q test), the pooled OR estimate of all studies was calculated by the fixed-effects model (the Mantel–Haenszel method) [46]. If homogeneity could not be assumed, a random-effects model (the DerSimonian and Laird method) was used [47]. Subgroup analyses were performed by cancer type, ethnicity, study design and sample size (i.e., no. of cases ≥150 vs. no. of cases <150). To verify the presence of potential publication bias, a standard error of log (OR) for each study was plotted against its log (OR). Funnel plot asymmetry was assessed by Egger’s linear regression test [48]. To assess the effect of individual studies on the overall risk of cancers, sensitivity analyses were performed by excluding each study individually and recalculating the ORs and 95% CI. The mRNA expression levels between the strata were assessed by using a Student’s *t* test, and the trend tests of transcript expression levels by genotypes were evaluated by using General linear model. This meta-analysis was performed using the software STATA version 10.0 (Stata Corporation, College Station, TX) and SAS software (version 9.1; SAS Institute, Cary, NC). All *P* values were two-sided, and a *P*<0.05 was considered statistically significant.

## Results

### Study characteristics

**Figure 2:**
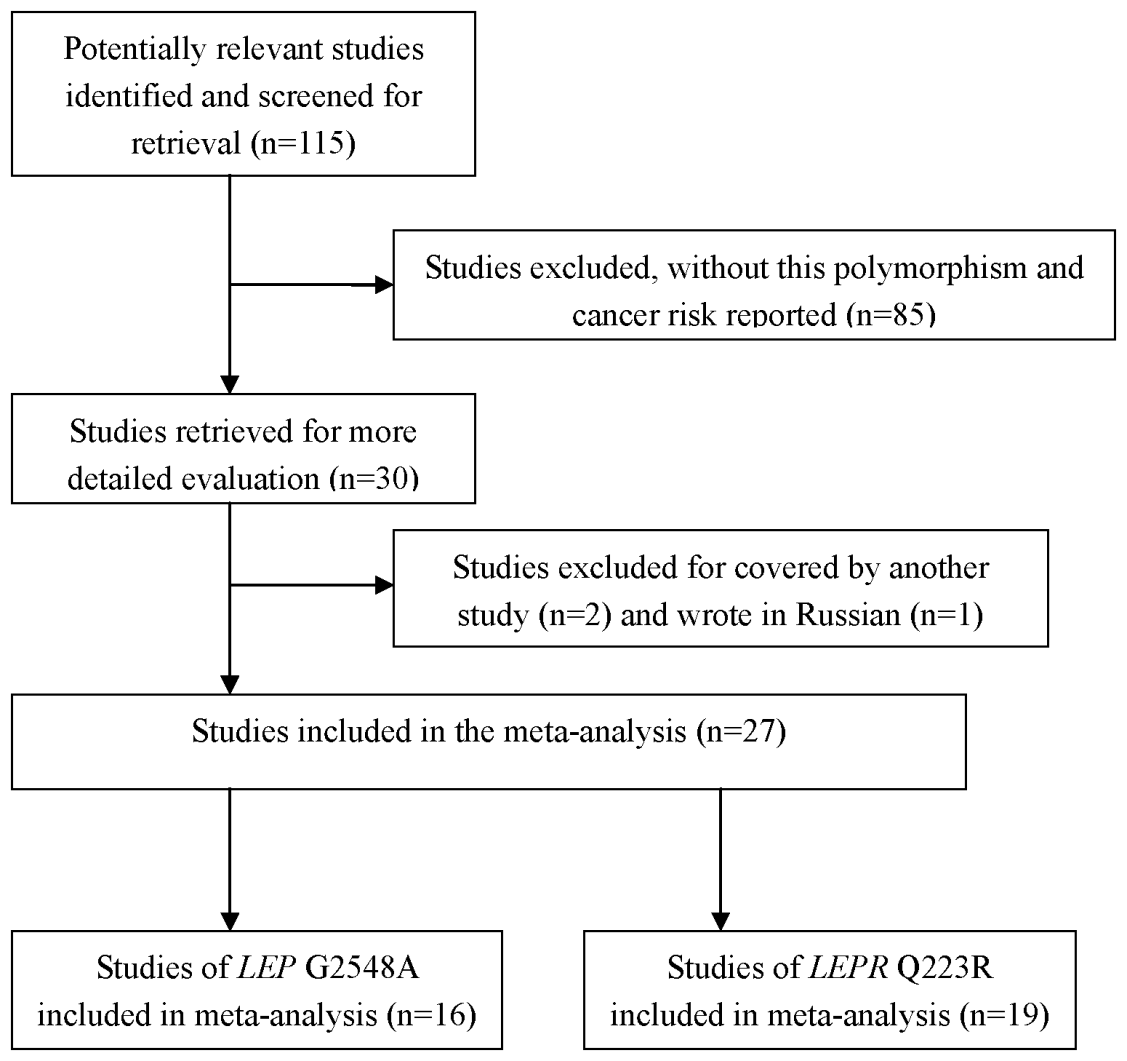
Flow chart of included studies for this meta-analysis.

As shown in Figure **2**, a total of 115 published records were retrieved, of which 85 were excluded after the abstracts were found to be irrelevant, and three papers were excluded, for two [49,50] of which were covered by another study [11], and one was written in Russian [26]. Finally, 27 papers met the inclusion criteria and were included in the meta-analysis (Table **1**). Overall, 16 studies with 6569 cases and 8405 controls investigated the *LEP* G2548A (or A19G) SNP, and another 19 studies with 7504 cases and 9581 controls investigated the *LEPR* Q223R SNP. The study of Teras et al. [29] on the two SNP was included only in the calculation of the dominant model, because the genotype distribution was not presented in enough detail.

**Table 1:** Characteristics of studies included in the meta-analysis.

Surname	Year	Country	Ethnicity	Cancer type	cases/controls	Source of controls	Genotype method	Polymorphisms
Kote-Jarai	2003	UK	Caucasian	Prostate cancer	273/262	PB*	PCR-RFLP	Q223R
Ribeiro	2004	Portugal	Caucasian	Prostate cancer	143/118	HB	PCR-RFLP	G2548A
Ribeiro	2006	Portugal	Caucasian	Lung cancer	102/342	HB	PCR-RFLP	G2548A
Woo	2006	Korea	Asian	Breast cancer	45/45	HB	PCR-sequencing	Q223R
Snoussi	2006	Tunisia	African	Breast cancer	308/222	HB	PCR-RFLP	G2548A, Q223R
Gallicchio	2007	USA	Caucasian	Breast cancer	53/872	PB	TaqMan	Q223R
Han	2008	China	Asian	Breast cancer	240/500	HB	PCR-RFLP	Q223R
Okobia	2008	Nigeria	African	Breast cancer	209/209	HB	PCR-RFLP	Q223R
Ulybina	2008	Russia	Caucasian	Breast cancer	110/105	HB	Real-time PCR	Q223R
Slattery	2008	USA	Mixed	Colorectal cancer	1565/1965	Mixed	TaqMan	G2548A
Doecke	2008	Australia	Caucasian	Esophageal cancer	261/1352	PB	Sequenom iPLEX	G2548A, Q223R
Ulybina	2008	Russia	Caucasian	Endometrial cancer	191/105	HB	Real-time PCR	Q223R
Teras	2009	USA	Caucasian	Breast cancer	641/650	PB	SNPstream	G2548A, Q223R
Moore	2009	Finland	Caucasian	Prostate cancer	947/863	PB	TaqMan	G2548A
Wang	2009	USA	Caucasian	Prostate cancer	253/257	PB	TaqMan	G2548A
Yapijakis	2009	Greece & Germany	Caucasian	Oral cancer	150/152	HB	PCR-RFLP	G2548A, Q223R
Pechlivanis	2009	Czech	Caucasian	Colorectal cancer	659/711	HB	TaqMan	G2548A, Q223R
Vašků	2009	Czech	Caucasian	Colorectal cancer	100/100	HB	PCR-sequencing	G2548A, Q223R
Tsilidis	2009	USA	Mixed	Colorectal cancer	204/362	PB	TaqMan	G2548A
Chovanec	2009	Czech	Caucasian	Endometrial cancer	66/66	HB	Unknown	G2548A
Cleveland	2010	USA	Caucasian	Breast cancer	1059/1101	PB	Unknown	G2548A, Q223R
Partida-Perez	2010	Mexico	Latin American	Colorectal cancer	68/102	HB	PCR-RFLP	G2548A
Dai	2010	China	Asian	Hepatocellular	82/102	HB	PCR-RFLP	Q223R
Nyante	2011	USA	Mixed	Breast cancer	1972/1775	PB	Illumina	Q223R
Kim	2012	Korea	Asian	Breast cancer	390/447	HB	MassARRAY	Q223R
Li	2012	China	Asian	Lung cancer	744/832	PB	PCR-RFLP	Q223R
Kim	2012	Korea	Asian	Gastric cancer	48/48	HB	PCR-RFLP	G2548A, Q223R

Notes: LEP G2548A is in high linkage disequilibrium with A19G; * Spouses of patients with CRC.

HB, Hospital based; PB, Population based; RFLP, Restriction fragment length polymorphisms polymerase chain reaction.

### Meta-analysis results

**Figure 3:**
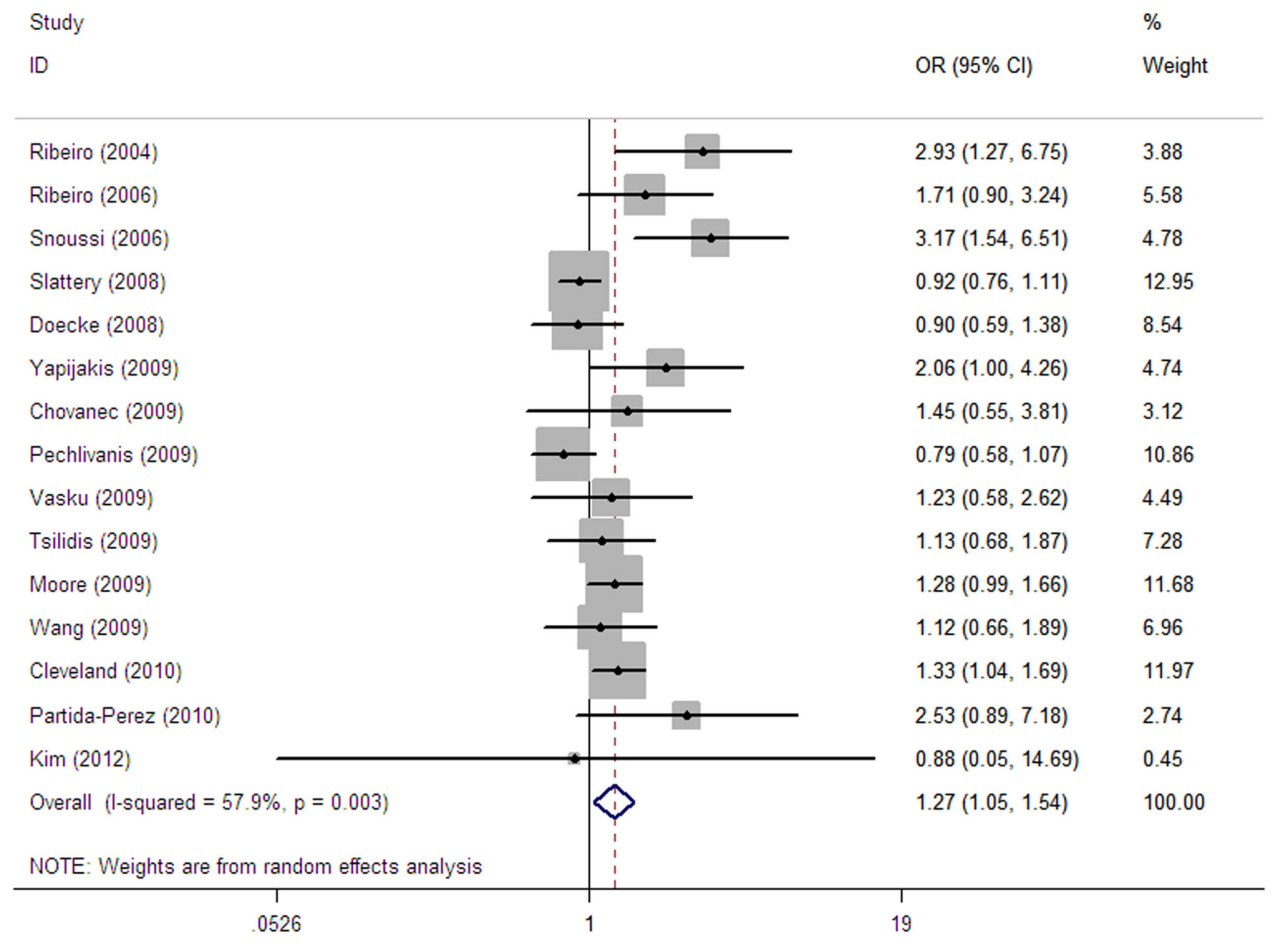
Forest plot for AA vs. GG of *LEP* G2548A polymorphism.

The overall results suggested a statistically significant association between *LEP* G2548A (or A19G) and risk of cancer (AA vs. GG: OR=1.27, 95% CI=1.05-1.54; AA vs. AG+GG: OR=1.19, 95% CI=1.00-1.41) (Table **2**, Figure **3**). In the subgroup analysis by ethnicity, a statistically significant association was found for Caucasians (AA vs. GG: OR=1.24, 95% CI=1.01-1.53; recessive model: OR=1.23, 95% CI=1.01-1.51) and Africans (AA vs. GG: OR=3.17, 95% CI=1.54-6.51; recessive model: OR=2.62, 95% CI=1.31-5.26) but not for other ethnic groups. In the subgroup analysis by tumor type, the *LEP* 2548A (or 19G) allele was significantly associated with risk of prostate cancer (AA vs. AG+GG: OR=1.26, 95%CI=1.05-1.51) but not with cancers of the breasts and colorectal or other specified cancers. In the subgroup analysis by sample size, a statistically significant association was found for studies with sample sizes <150 (AA vs. GG: OR=1.78, 95% CI=1.24-2.54; AA vs. AG+GG: OR=1.33, 95% CI=1.00-1.78) but not for those with sample sizes ≥150.

**Table 2:** Meta-analysis of the association between *LEP* G2548A polymorphism and cancer risk.

Variables	No. of studies^a^	Homozygous co-dominant	*P*_het_^b^	Heterozygous co-dominant	*P*_het_^b^	Recessive	*P*_het_^b^	Dominant	*P*_het_^b^
		AA vs. GG		AG vs. GG		(AA vs. AG+GG)		(AA+AG vs. GG)	
All	15	1.27 (1.05-1.54)	0.003	1.04 (0.96-1.13)	0.154	1.19 (1.00-1.41)	0.000	1.08 (0.97-1.20)	0.089
Cancer type									
Breast	2	1.91 (0.82-4.45)	0.025	1.02 (0.86-1.21)	0.033	1.74 (0.96-3.17)	0.088	1.12 (0.85-1.46)	0.030
Colorectal	5	0.97 (0.78-1.20)	0.216	1.03 (0.92-1.17)	0.188	0.92 (0.81-1.05)	0.532	1.02 (0.84-1.23)	0.160
Prostate	3	1.42 (0.94-2.12)	0.138	1.13 (0.94-1.36)	0.068	1.26 (1.05-1.51)	0.501	1.30 (0.92-1.84)	0.060
Others	5	1.32 (0.91-1.92)	0.270	0.96 (0.74-1.24)	0.736	1.27 (0.79-2.08)	0.004	1.02 (0.80-1.32)	0.952
Ethnicity									
Caucasian	10	1.24 (1.01-1.53)	0.036	0.99 (0.89-1.10)	0.333	1.23 (1.01-1.51)	0.003	1.03 (0.93-1.15)	0.299
Latin American	1	2.53 (0.89-7.18)	/	2.97 (1.17-7.50)	/	1.08 (0.53-2.21)	/	2.83 (1.15-6.98)	/
African	1	3.17 (1.54-6.51)	/	1.45 (1.01-2.07)	/	2.62 (1.31-5.26)	/	1.62 (1.14-2.29)	/
Asian	1	0.88 (0.05-14.69)		1.29 (0.07-22.42)		0.69 (0.30-1.61)		1.00 (0.06-16.46)	/
Mixed	2	0.94 (0.79-1.13)	0.455	1.05 (0.91-1.22)	0.796	0.96 (0.76-1.19)	0.213	1.02 (0.89-1.17)	0.899
Source of controls									
Hospital	9	1.70 (1.10-2.61)	0.002	1.10 (0.94-1.29)	0.028	1.39 (0.99-1.95)	0.003	1.28 (0.97-1.69)	0.011
Population	5	1.22 (1.05-1.41)	0.604	0.99 (0.88-1.13)	0.894	1.15 (0.95-1.38)	0.074	1.03 (0.93-1.15)	0.923
Mixed	1	0.92 (0.76-1.11)	/	1.06 (0.91-1.23)	/	0.89 (0.75-1.05)	/	1.02 (0.88-1.17)	/
Sample size in cases									
< 150	6	1.78 (1.24-2.54)	0.677	1.29 (0.98-1.71)	0.060	1.33 (1.00-1.78)	0.397	1.44 (0.98-2.13)	0.120
>=150	9	1.16 (0.95-1.43)	0.003	1.02 (0.94-1.11)	0.588	1.16 (0.95-1.41)	0.000	1.03 (0.95-1.12)	0.360

^a^ Only presented the study with enough detail, one study was included only in the calculation of the dominant model.

^b^ *P* value of the Q-test for heterogeneity test.

**Figure 4:**
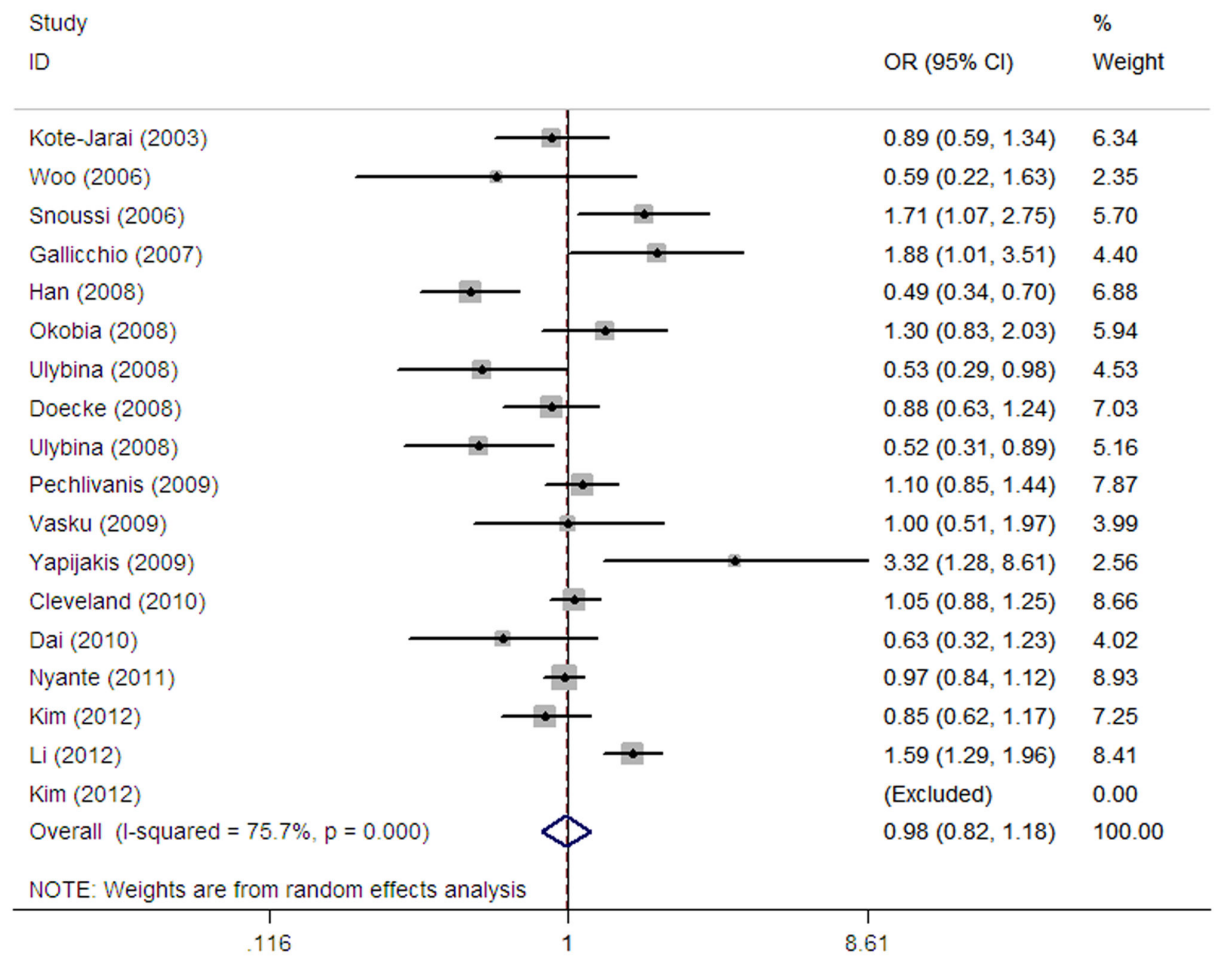
Forest plot for the *LEPR* Q223R polymorphism (recessive model).

For the *LEPR* Q223R SNP, no statistically significant association with cancer risk was found (Table **3**, Figure **4**). In the stratified analysis by ethnicity, however, a statistically significant association was observed for Africans (GG vs. AA: OR=1.85, 95% CI=1.23-2.79; GA vs. AA: OR=1.48, 95% CI=1.08-2.01; GG vs. GA+AA: OR=1.48, 95% CI=1.07-2.05; GG+GA vs. AA: OR=1.58, 95% CI=1.14-2.20), but not for Caucasians and other ethnic populations. No statistically significant association was found in the further stratification by tumor type, source of controls, and sample size.

**Table 3:** Meta-analysis of the association between *LEPR* Q223R polymorphism and cancer risk.

Variables	No. of studies^a^	Homozygous co-dominant	*P*_het_^b^	Heterozygous co-dominant	*P*_het_^b^	Recessive	*P*_het_^b^	Dominant	*P*_het_^b^
		GG vs. AA		AG vs. AA		(GG vs. AG+AA)		(GG+AG vs. AA)	
All	18	1.02 (0.76-1.39)	0.000	1.08 (0.88-1.34)	0.000	0.98 (0.82-1.18)	0.000	1.03 (0.83-1.29)	0.000
Cancer type									
Breast	9	0.94 (0.62-1.42)	0.000	0.97 (0.72-1.31)	0.000	0.95 (0.76-1.20)	0.000	0.93 (0.70-1.24)	0.000
Colorectal	2	1.15 (0.86-1.53)	0.507	1.25 (0.70-2.23)	0.090	1.09 (0.85-1.39)	0.789	1.23 (0.76-1.98)	0.130
Prostate	1	0.82 (0.52-1.29)	/	0.85 (0.58-1.26)	/	0.89 (0.59-1.34)	/	0.84 (0.59-1.19)	/
Others	6	1.09 (0.49-2.39)	0.000	1.27 (0.83-1.94)	0.029	1.04 (0.62-1.75)	0.000	1.17 (0.90-1.96)	0.000
Ethnicity									
Caucasian	9	1.08 (0.84-1.40)	0.020	1.10 (0.96-1.26)	0.363	0.98 (0.78-1.24)	0.006	1.06 (0.91-1.23)	0.075
East Asian	6	0.44 (0.08-2.48)	0.000	0.49 (0.11-2.13)	0.000	0.79 (0.46-1.36)	0.000	0.44 (0.09-2.33)	0.000
African	2	1.85 (1.23-2.79)	0.275	1.48 (1.08-2.01)	0.302	1.48 (1.07-2.05)	0.403	1.58 (1.14-2.20)	0.245
Mixed	1	0.91 (0.76-1.09)	/	0.92 (0.78-1.08)	/	0.97 (0.84-1.12)	/	0.92 (0.79-1.06)	/
Source of controls									
Hospital	12	0.86 (0.49-1.51)	0.000	0.99 (0.69-1.43)	0.000	0.89 (0.66-1.19)	0.000	0.90 (0.60-1.38)	0.000
Population	6	1.22 (0.84-1.76)	0.000	1.14 (0.86-1.51)	0.000	1.12 (0.90-1.39)	0.001	1.11 (0.84-1.46)	0.000
Sample size in cases									
< 150	6	0.89 (0.37-2.12)	0.014	1.08 (0.55-2.13)	0.046	0.84 (0.51-1.38)	0.040	1.03 (0.53-2.01)	0.034
>=150	12	1.04 (0.75-1.46)	0.000	1.07 (0.85-1.35)	0.000	1.02 (0.84-1.24)	0.000	1.02 (0.80-1.31)	0.000

^a^ Only presented the study with enough detail, one study was included only in the calculation of the dominant model.

^b^ *P* value of the Q-test for heterogeneity test.

### The mRNA expression by genotypes

The mRNA expression levels of *LEP* and *LEPR* by the genotypes for four ethnicities are shown in Table **4**. We did not find any differences in mRNA expression by genotypes among different ethnicities. No trend of transcript expression levels by genotypes was found for *LEP* or *LEPR*. These data suggest that the variants under investigation may not have a significant effect on gene expression, at least at mRNA levels.

**Table 4:** *LEP* and *LEPR* mRNA expression by the genotypes of SNPs, using data from the HapMap ^**a**^.

Populations	*LEP* rs7799039 (G2548A)					*LEPR* rs1137101 (Q223R)				
	Genotypes	No.	Mean ± SD	*P* ^b^	*P*_trend_^c^	Genotypes	No.	Mean ± SD	*P* ^b^	*P*_trend_^c^
CHB	GG	4	8.82±0.22		0.913	GG	36	8.70±0.23		0.819
	AG	15	8.66±0.23	0.229		AG	8	8.78±0.22	0.402	
	AA	26	8.73±0.23	0.422		AA	1	8.53	0.468	
	AG/AA	41	8.70±0.23	0.311		AG/AA	9	8.75±0.22	0.575	
JPT ^d^	GG	1	8.41		0.653	GG	32	8.52±0.22		0.774
	AG	18	8.51±0.24	0.688		AG	12	8.50±0.15	0.774	
	AA	26	8.53±0.20	0.562		AA	0	-	-	
	AG/AA	44	8.52±0.21	0.609		AG/AA	12	8.50±0.15	0.774	
CEU ^d^	GG	20	8.53±0.30		0.473	GG	26	8.49±0.27		0.427
	AG	44	8.45±0.24	0.228		AG	44	8.48±0.23	0.902	
	AA	21	8.47±0.25	0.492		AA	19	8.43±0.22	0.421	
	AG/AA	65	8.45±0.24	0.242		AG/AA	63	8.46±0.23	0.674	
YRI ^d^	GG	87	8.57±0.24		0.749	GG	31	8.57±0.27		0.698
	AG	2	8.62±0.05	0.749		AG	46	8.59±0.22	0.728	
	AA	0	-	-		AA	12	8.51±0.26	0.561	
	AG/AA	2	8.62±0.05	0.749		AG/AA	58	8.57±0.22	0.936	

^a^ Genotyping data and mRNA expression levels for *LEP* or *LEPR* by genotypes were obtained from the HapMap phase II release 23 data from EBV-transformed lymphoblastoid cell lines from 270 individuals, including 45 unrelated Han Chinese in Beijing (CHB).

^b^ Two-side Student’s *t* test within the stratum.

^c^ *P* values for the trend test of mRNA expression among three genotypes for each SNP from a general linear model.

^d^ There were missing data because genotyping data for six individuals were not available for *LEP* and three individuals were not available for *LEPR.*

### Publication bias

For *LEP* G2548A (or A19G), there was evidence for publication bias under a homozygous additive model (the Egger’s test: AA vs. GG: *P*=0.034); however, this was not observed under other genetic models (AG vs. GG: *P*=0.174; recessive model: *P*=0.138; dominant model: *P*=0.071). The publication bias may be ascribed to small sample sizes the included studies had. When studies with cases smaller than 150 in numbers were excluded, the publication bias disappeared, but the significant association also disappeared.

No publication bias was detected for *LEPR* Q223R (the Egger’s test: GG vs. AA: *P*=0.559, AG vs. AA: *P*=0.686, recessive model: *P*=0.600, dominant model: *P*=0.600).

## Discussion

It is well recognized that individual susceptibility to cancer varies, even with the same environmental exposure. Therefore, a role for genetic variation, such as SNPs of genes involved in carcinogenesis, has been suggested. Epidemiological studies have shown that overweight and obesity might be associated with an increased risk of cardiovascular disease and type II diabetes; moreover, excessive body weight has been directly associated with risk of cancer at several organ sites, including the colon, breasts (in postmenopausal women), endometrium, esophagus, and kidney [51]. Formerly, immune dysfunction has been shown to be associated with obesity [20], whereas leptin concentrations were recently found to be higher in Africans, compared with Caucasians, after adjustment for BMI and other factors [52].

The Q223R SNP (but not the K109R or K656N SNPs) of the *LEPR* gene has been reported to be associated with obesity and to predict a small percentage of body weight and body composition variability in a genetically homogeneous population [8]. Previous reports demonstrated that genetic variation in *LEPR* affected cancer susceptibility with significantly higher frequency of the *LEPR* 223Arg allele in patients than in controls [9,11,22,24,33]; however, this association was not be replicated by later studies [14,16,19,23,31,36]. Likewise, previous reports also demonstrated that *LEP* 2548AA was associated with an increased risk of cancer [12,13,20,21,33,34]; however, replication of this finding by others also failed as well [25,28,31].

In this meta-analysis, we found statistical evidence for a significant but week association of cancer risk with the *LEP* G2548A (or A19G) SNP but not with the *LEPR* Q223R SNP. There are several biologically plausible explanations for this finding. Firstly, it has been described that genetic variants in the promoter region of *LEP* can influence leptin expression, possibly at the transcriptional level, thereby altering adipose secretion levels of the hormone [17]. Additionally, it is also likely that the observed association may be due to improved study power from pooling studies with small sample sizes that separately may have had insufficient statistical power to detect a weak effect. Thus, in the genotype-based mRNA expression analysis using data from HapMap for the *LEP* G2548A, we did not find statistical difference may be ascribed to small sample size for each ethnicity or the G2548A may have a weak effect. In the subgroup analysis by tumor type, an observed association between *LEP* 2548A (or 19G) and risk of prostate cancer suggests that this SNP may be disease specific, because all the prostate cancer patients were Caucasian decent. In the subgroup analysis by sample size, we found that the association between studies with small sample sizes and cancer risk for the G2548A polymorphism may be ascribed to some selection bias. In contrast to another meta-analysis, however, we were not able to find a statistically significant association between *LEPR* Q223R and risk of breast cancer [53]. This could be explained by the fact that we included a latest study on breast cancer that included 1972 cases and 1775 controls, a null study that was not included in the previous meta-analysis.

In exploring possible functional relevance of the SNPs under investigation, we did not find any differences in or trends of the mRNA expression levels of *LEP* and *LEPR* by their genotypes in four ethnic groups. Cancer is a complex and multifactorial disease, and gene-gene and gene-environment interactions may contribute greatly to its occurrence, but a single nucleotide alteration may be insufficient to alter the mRNA expression, even for those SNPs in the coding regions that may lead to amino acid change or the polymorphisms in a promoter may have a subtle, potential effect on the gene expression.

Though we performed this meta-analysis using the pooled data that can yield more reliable or statistically more powerful results, several limitations should be addressed. First, significant heterogeneity were found for both of these two polymorphisms that may influence the interpretation of the results. Second, the individual sample sizes for cases of most studies included in the analysis were relatively small (<500) except for seven studies [25,27-29,34,36,39], and there were only one study based on the population of Latin Americans, Africans and Asians for the G2548A polymorphism, respectively, which did not provide insufficient statistical power to investigate the real association. Third, most of the studies used hospital-based controls that may result in some selection biases. Finally, the lacking of original data such as age, sex, smoking and drinking status, BMI, environmental factors and other lifestyle, limited our ability to further evaluate of gene-gene and gene-environment interactions.

In conclusion, this meta-analysis found that the *LEP* 2548AA genotype was associated with a weakly increased risk of cancer, mainly for prostate cancer, while *LEPR* Q223R was not. However, given the relatively limited sample sizes and the lack of detailed information, this analysis with mixed ethnicities was not able to address cancer outcomes and biological evidence for genotype-phenotype (mRNA expression) correlations. It is clear that further studies are warranted to validate the association between the *LEP* G2548A polymorphism and cancer risk.

## Supporting Information

Checklist S1 (DOC)

## References

[1] Jemal A, Bray F, Center MM, Ferlay J, Ward E et al. (2011) Global cancer statistics. CA Cancer J Clin 61: 69-90. doi:10.3322/caac.20107. PubMed: 21296855. 21296855

[2] Lichtenstein P, Holm NV, Verkasalo PK, Iliadou A, Kaprio J et al. (2000) Environmental and heritable factors in the causation of cancer--analyses of cohorts of twins from Sweden, Denmark, and Finland. N Engl J Med 343: 78-85. doi:10.1056/NEJM200007133430201. PubMed: 10891514. 10891514

[3] Lichtenstein P, Holm NV, Verkasalo PK, Iliadou A, Kaprio J (2000) Environmental and heritable factors in the causation of cancer: analyses of cohorts of twins from Sweden, Denmark, and Finland, N Engl J Med 343:78-84. 10891514 10.1056/NEJM200007133430201

[4] Friedman JM, Halaas JL (1998) Leptin and the regulation of body weight in mammals. Nature 395: 763-770. doi:10.1038/27376. PubMed: 9796811. 9796811

[5] Unger RH, Zhou YT, Orci L (1999) Regulation of fatty acid homeostasis in cells: novel role of leptin. Proc Natl Acad Sci U S A 96: 2327-2332. doi:10.1073/pnas.96.5.2327. PubMed: 10051641. 10051641 PMC26783

[6] Lakka HM, Oksanen L, Tuomainen TP, Kontula K, Salonen JT (2000) The common pentanucleotide polymorphism of the 3'-untranslated region of the leptin receptor gene is associated with serum insulin levels and the risk of type 2 diabetes in non-diabetic men: a prospective case-control study. J Intern Med 248: 77-83. doi:10.1046/j.1365-2796.2000.00696.x. PubMed: 10947884. 10947884

[7] Lönnqvist F, Arner P, Nordfors L, Schalling M (1995) Overexpression of the obese (ob) gene in adipose tissue of human obese subjects. Nat Med 1: 950-953. doi:10.1038/nm0995-950. PubMed: 7585223. 7585223

[8] Yiannakouris N, Yannakoulia M, Melistas L, Chan JL, Klimis-Zacas D et al. (2001) The Q223R polymorphism of the leptin receptor gene is significantly associated with obesity and predicts a small percentage of body weight and body composition variability. J Clin Endocrinol Metab 86: 4434-4439. doi:10.1210/jc.86.9.4434. PubMed: 11549688. 11549688

[9] Snoussi K, Strosberg AD, Bouaouina N, Ben Ahmed S, Helal AN et al. (2006) Leptin and leptin receptor polymorphisms are associated with increased risk and poor prognosis of breast carcinoma. BMC Cancer 6: 38. doi:10.1186/1471-2407-6-38. PubMed: 16504019. 16504019 PMC1397853

[10] Liu CL, Chang YC, Cheng SP, Chern SR, Yang TL et al. (2007) The roles of serum leptin concentration and polymorphism in leptin receptor gene at codon 109 in breast cancer. Oncology 72: 75-81. doi:10.1159/000111097. PubMed: 18004080. 18004080

[11] Han CZ, Du LL, Jing JX, Zhao XW, Tian FG et al. (2008) Associations among lipids, leptin, and leptin receptor gene Gin223Arg polymorphisms and breast cancer in China. Biol Trace Elem Res 126: 38-48. doi:10.1007/s12011-008-8182-z. PubMed: 18668212. 18668212

[12] Ribeiro R, Vasconcelos A, Costa S, Pinto D, Morais A et al. (2004) Overexpressing leptin genetic polymorphism (-2548 G/A) is associated with susceptibility to prostate cancer and risk of advanced disease. Prostate 59: 268-274. doi:10.1002/pros.20004. PubMed: 15042602. 15042602

[13] Ribeiro R, Araújo AP, Coelho A, Catarino R, Pinto D et al. (2006) A functional polymorphism in the promoter region of leptin gene increases susceptibility for non-small cell lung cancer. Eur J Cancer 42: 1188-1193. doi:10.1016/j.ejca.2006.02.004. PubMed: 16630717. 16630717

[14] Chia VM, Newcomb PA, Lampe JW, White E, Mandelson MT et al. (2007) Leptin concentrations, leptin receptor polymorphisms, and colorectal adenoma risk. Cancer Epidemiol Biomarkers Prev 16: 2697-2703. doi:10.1158/1055-9965.EPI-07-0467. PubMed: 18086776. 18086776

[15] Mantzoros CS, Moschos SJ (1998) Leptin: in search of role(s) in human physiology and pathophysiology. Clin Endocrinol (Oxf) 49: 551-567. doi:10.1046/j.1365-2265.1998.00571.x. PubMed: 10197068. 10197068

[16] Woo HY, Park H, Ki CS, Park YL, Bae WG (2006) Relationships among serum leptin, leptin receptor gene polymorphisms, and breast cancer in Korea. Cancer Lett 237: 137-142. doi:10.1016/j.canlet.2005.05.041. PubMed: 16011872. 16011872

[17] Hoffstedt J, Eriksson P, Mottagui-Tabar S, Arner P (2002) A polymorphism in the leptin promoter region (-2548 G/A) influences gene expression and adipose tissue secretion of leptin. Horm Metab Res 34: 355-359. doi:10.1055/s-2002-33466. PubMed: 12189581. 12189581

[18] Chovanec J, Bienertová-Vasků JA, Dostálová Z (2009) Leptin--2548 g/A polymorphism in endometrial cancer. Klin Onkol 22: 223-227. PubMed: 19886360. 19886360

[19] Kote-Jarai Z, Singh R, Durocher F, Easton D, Edwards SM et al. (2003) Association between leptin receptor gene polymorphisms and early-onset prostate cancer. BJU Int 92: 109-112. doi:10.1046/j.1464-410X.2003.04272.x. PubMed: 12823393. 12823393

[20] Skibola CF, Holly EA, Forrest MS, Hubbard A, Bracci PM et al. (2004) Body mass index, leptin and leptin receptor polymorphisms, and non-hodgkin lymphoma. Cancer Epidemiol Biomarkers Prev 13: 779-786. PubMed: 15159310. 15159310

[21] Willett EV, Skibola CF, Adamson P, Skibola DR, Morgan GJ et al. (2005) Non-Hodgkin's lymphoma, obesity and energy homeostasis polymorphisms. Br J Cancer 93: 811-816. doi:10.1038/sj.bjc.6602762. PubMed: 16160698. 16160698 PMC2361643

[22] Gallicchio L, McSorley MA, Newschaffer CJ, Huang HY, Thuita LW et al. (2007) Body mass, polymorphisms in obesity-related genes, and the risk of developing breast cancer among women with benign breast disease. Cancer Detect Prev 31: 95-101. doi:10.1016/j.cdp.2007.02.004. PubMed: 17428620. 17428620

[23] Doecke JD, Zhao ZZ, Stark MS, Green AC, Hayward NK et al. (2008) Single nucleotide polymorphisms in obesity-related genes and the risk of esophageal cancers. Cancer Epidemiol Biomarkers Prev 17: 1007-1012. doi:10.1158/1055-9965.EPI-08-0023. PubMed: 18398047. 18398047

[24] Okobia MN, Bunker CH, Garte SJ, Zmuda JM, Ezeome ER et al. (2008) Leptin receptor Gln223Arg polymorphism and breast cancer risk in Nigerian women: a case control study. BMC Cancer 8: 338. doi:10.1186/1471-2407-8-338. PubMed: 19017403. 19017403 PMC2613914

[25] Slattery ML, Wolff RK, Herrick J, Caan BJ, Potter JD (2008) Leptin and leptin receptor genotypes and colon cancer: gene-gene and gene-lifestyle interactions. Int J Cancer 122: 1611-1617. PubMed: 18059035. 18059035 10.1002/ijc.23135 PMC2430084

[26] Ulybina IuM, Imianitov EN, Vasil'ev DA, Bershtein LM (2008) [Polymorphism of glucose intolerance and insulin resistance susceptibility genes in oncological patients]. Mol Biol (Mosk) 42: 947-956. 19140314

[27] Moore SC, Leitzmann MF, Albanes D, Weinstein SJ, Snyder K et al. (2009) Adipokine genes and prostate cancer risk. Int J Cancer 124: 869-876. doi:10.1002/ijc.24043. PubMed: 19035456. 19035456 PMC2879625

[28] Pechlivanis S, Bermejo JL, Pardini B, Naccarati A, Vodickova L et al. (2009) Genetic variation in adipokine genes and risk of colorectal cancer. Eur J Endocrinol 160: 933-940. doi:10.1530/EJE-09-0039. PubMed: 19273568. 19273568

[29] Teras LR, Goodman M, Patel AV, Bouzyk M, Tang W et al. (2009) No association between polymorphisms in LEP, LEPR, ADIPOQ, ADIPOR1, or ADIPOR2 and postmenopausal breast cancer risk. Cancer Epidemiol Biomarkers Prev 18: 2553-2557. doi:10.1158/1055-9965.EPI-09-0542. PubMed: 19723917. 19723917

[30] Tsilidis KK, Helzlsouer KJ, Smith MW, Grinberg V, Hoffman-Bolton J et al. (2009) Association of common polymorphisms in IL10, and in other genes related to inflammatory response and obesity with colorectal cancer. Cancer Causes Control 20: 1739-1751. doi:10.1007/s10552-009-9427-7. PubMed: 19760027. 19760027 PMC4119174

[31] Vasků A, Vokurka J, Bienertová-Vasků J (2009) Obesity-related genes variability in Czech patients with sporadic colorectal cancer: preliminary results. Int J Colorectal Dis 24: 289-294. doi:10.1007/s00384-008-0553-6. PubMed: 18704460. 18704460

[32] Wang MH, Helzlsouer KJ, Smith MW, Hoffman-Bolton JA, Clipp SL et al. (2009) Association of IL10 and other immune response- and obesity-related genes with prostate cancer in CLUE II. Prostate 69: 874-885. doi:10.1002/pros.20933. PubMed: 19267370. 19267370 PMC3016874

[33] Yapijakis C, Kechagiadakis M, Nkenke E, Serefoglou Z, Avgoustidis D et al. (2009) Association of leptin -2548G/A and leptin receptor Q223R polymorphisms with increased risk for oral cancer. J Cancer Res Clin Oncol 135: 603-612. doi:10.1007/s00432-008-0494-z. PubMed: 18855010. 18855010 PMC12160154

[34] Cleveland RJ, Gammon MD, Long CM, Gaudet MM, Eng SM et al. (2010) Common genetic variations in the LEP and LEPR genes, obesity and breast cancer incidence and survival. Breast Cancer Res Treat 120: 745-752. doi:10.1007/s10549-009-0503-1. PubMed: 19697123. 19697123 PMC3571680

[35] Partida-Pérez M, de la Luz Ayala-Madrigal M, Peregrina-Sandoval J, Macías-Gómez N, Moreno-Ortiz J et al. (2010) Association of LEP and ADIPOQ common variants with colorectal cancer in Mexican patients. Cancer Biomark 7: 117-121. PubMed: 21263187. 21263187 10.3233/CBM-2010-0154 PMC12922883

[36] Nyante SJ, Gammon MD, Kaufman JS, Bensen JT, Lin DY et al. (2011) Common genetic variation in adiponectin, leptin, and leptin receptor and association with breast cancer subtypes. Breast Cancer Res Treat 129: 593-606. doi:10.1007/s10549-011-1517-z. PubMed: 21516303. 21516303 PMC3355661

[37] Kim EY, Chin HM, Park SM, Jeon HM, Chung WC et al. (2012) Susceptibility of gastric cancer according to leptin and leptin receptor gene polymorphisms in Korea. J Korean Surg Soc 83: 7-13. doi:10.4174/jkss.2012.83.1.7. PubMed: 22792528. 22792528 PMC3392320

[38] Kim KZ, Shin A, Lee YS, Kim SY, Kim Y et al. (2012) Polymorphisms in adiposity-related genes are associated with age at menarche and menopause in breast cancer patients and healthy women. Hum Reprod 27: 2193-2200. doi:10.1093/humrep/des147. PubMed: 22537818. 22537818

[39] Li Y, Geng J, Wang Y, Lu Q, Du Y et al. (2012) The role of leptin receptor gene polymorphisms in determining the susceptibility and prognosis of NSCLC in Chinese patients. J Cancer Res Clin Oncol 138: 311-316. doi:10.1007/s00432-011-1098-6. PubMed: 22127368. 22127368 PMC11824543

[40] Dai K, Chen J, Yang L, Gong Z (2010) The relationship of serum leptin and leptin receptor polymorphisms with primary hepatocellular carcinoma. Chin J Gastroenterol Hepatol 19: 722-724.

[41] Holm K, Melum E, Franke A, Karlsen TH (2010) SNPexp - A web tool for calculating and visualizing correlation between HapMap genotypes and gene expression levels. BMC Bioinformatics 11: 600. doi:10.1186/1471-2105-11-600. PubMed: 21167019. 21167019 PMC3022629

[42] International HapMap Consortium (2003) The International HapMap Project. Nature 426: 789-796. doi:10.1038/nature02168. PubMed: 14685227. 14685227

[43] He J, Qiu LX, Wang MY, Hua RX, Zhang RX et al. (2012) Polymorphisms in the XPG gene and risk of gastric cancer in Chinese populations. Hum Genet 131: 1235-1244. doi:10.1007/s00439-012-1152-8. PubMed: 22371296. 22371296

[44] Stranger BE, Forrest MS, Dunning M, Ingle CE, Beazley C et al. (2007) Relative impact of nucleotide and copy number variation on gene expression phenotypes. Science 315: 848-853. doi:10.1126/science.1136678. PubMed: 17289997. 17289997 PMC2665772

[45] He J, Shi TY, Zhu ML, Wang MY, Li QX et al. (2013) Associations of Lys939Gln and Ala499Val polymorphisms of the XPC gene with cancer susceptibility: A meta-analysis. Int J Cancer, 133: 1765–75. doi:10.1002/ijc.28089. PubMed: 23400628. 23400628

[46] Mantel N, Haenszel W (1959) Statistical aspects of the analysis of data from retrospective studies of disease. J Natl Cancer Inst 22: 719-748. PubMed: 13655060. 13655060

[47] DerSimonian R, Laird N (1986) Meta-analysis in clinical trials. Control Clin Trials 7: 177-188. doi:10.1016/0197-2456(86)90046-2. PubMed: 3802833. 3802833

[48] Egger M, Davey Smith G, Schneider M, Minder C (1997) Bias in meta-analysis detected by a simple, graphical test. BMJ 315: 629-634. doi:10.1136/bmj.315.7109.629. PubMed: 9310563. 9310563 PMC2127453

[49] Han CZ, Shi J, Du LL, Jing JX, Zhao XW et al. (2007) [Association among lipids, leptin and leptin receptor polymorphisms with risk of breast cancer]. Zhonghua Liu Xing Bing Xue Za Zhi 28: 136-140. PubMed: 17649682. 17649682

[50] Han CZ, Du LL, Jing JX, Zhao XW, Tian FG et al. (2011) [Relationship between the mutation of leptin receptor gene and tumorigenesis of breast cancer]. Zhonghua Zhong Liu Za Zhi 33: 207-211. PubMed: 21575521. 21575521

[51] Bianchini F, Kaaks R, Vainio H (2002) Overweight, obesity, and cancer risk. Lancet Oncol 3: 565-574. doi:10.1016/S1470-2045(02)00849-5. PubMed: 12217794. 12217794

[52] Cohen SS, Fowke JH, Cai Q, Buchowski MS, Signorello LB et al. (2012) Differences in the Association between Serum Leptin Levels and Body Mass Index in Black and White Women: A Report from the Southern Community Cohort Study. Ann Nutr Metab 60: 90-97. doi:10.1159/000336180. PubMed: 22353927. 22353927 PMC3710998

[53] He BS, Pan YQ, Zhang Y, Xu YQ, Wang SK (2012) Effect of LEPR Gln223Arg polymorphism on breast cancer risk in different ethnic populations: a meta-analysis. Mol Biol Rep 39: 3117-3122. doi:10.1007/s11033-011-1076-8. PubMed: 21698367. 21698367

